# Social Exclusion Modifies Climate and Deforestation Impacts on a Vector-Borne Disease

**DOI:** 10.1371/journal.pntd.0000176

**Published:** 2008-02-06

**Authors:** Luis Fernando Chaves, Justin M. Cohen, Mercedes Pascual, Mark L. Wilson

**Affiliations:** 1 Department of Ecology and Evolutionary Biology, University of Michigan, Ann Arbor, Michigan, United States of America; 2 Department of Epidemiology, School of Public Health, University of Michigan, Ann Arbor, Michigan, United States of America; Harvard University, United States of America

## Abstract

**Background:**

The emergence of American Cutaneous Leishmaniasis (ACL) has been associated with changes in the relationship between people and forests, leading to the view that forest ecosystems increase infection risk and subsequent proposal that deforestation could reduce re-emergence of this disease.

**Methodology/Principal Findings:**

We analyzed county-level incidence rates of ACL in Costa Rica (1996–2000) as a function of social and environmental variables relevant to transmission ecology with statistical models that incorporate breakpoints. Once social marginality was taken into account, the effect of living close to a forest on infection risk was small, and diminished exponentially above a breakpoint. Forest cover was associated with the modulation of temporal effects of El Niño Southern Oscillation (ENSO) at small spatial scales, revealing an additional complex interplay of environmental forces and disease patterns.

**Conclusions/Significance:**

Social factors, which previously have not been evaluated rigorously together with environmental and climatic factors, appear to play a critical role that may ultimately determine disease risk.

## Author Summary

American Cutaneous Leishmaniasis emergence has been associated with changes in the interaction between people and forests. The association between outbreaks and forest clearance, higher risk for populations living close to forests, and the absence of this disease from urban settings has led to the proposal that it will disappear with the destruction of primary forests. This view ignores the complex nature of deforestation as a product of socioeconomic inequities. Our study shows that such inequities, as measured by a marginalization index, may ultimately determine risk within the country, with socially excluded populations most affected by the disease. Contrary to the established view, living close to the forest edge can diminish the risk provided other factors are taken into account. Additionally, differences in vulnerability to climatic variability appear to interact with forest cover to influence risk across counties where the disease has its largest burden. Incidence exacerbation associated with El Niño Southern Oscillation is observed in counties with larger proportions of deforestation. Our study calls for control efforts targeted to socially excluded populations and for more localized ecological studies of transmission in vectors and reservoirs in order to understand the role of biodiversity changes in driving the emergence of this disease.

## Introduction

American cutaneous leishmaniasis (ACL), a neglected infectious disease [1]–[4], is one of the main emerging and re-emerging vector-borne diseases in the Americas. It is a zoonotic vector-borne disease, caused by several species of *Leishmania* (Kinetoplastida: Trypanosomatidae) parasites and transmitted by sand flies (Diptera: Psychodidae). The (re)emergence of ACL has been associated with deforestation in the neotropics. For example, infection is highest among people living close to forest edges [5],[6], and also elevated among workers that extract natural resources in forested areas [7],[8]. This association with forest proximity/deforestation has led to the view that large-scale landscape transformation may reduce ACL emergence [6],[8],[9]. However, studies of ACL and forest cover thus far have ignored the multidimensionality of factors that shape patterns of infectious diseases [10]. Such multidimensionality is underscored by Schmalhausen's law, which states that biological systems at the boundary of their tolerance along any dimension of existence become more vulnerable to small changes along other such dimensions [11]. We suggest that this general principle is relevant to understanding environmental change and infectious diseases, and more generally ecosystem functioning and diversity conservation, given the interactions of these phenomena with social and economic realms.

Here we examined county-level ACL case data from 1996 through 2000 for Costa Rica, a country that proportionally has had the largest rate of landscape transformation in the New World [12]–[14], and report results contrary to the perspective that forest cover is the major risk factor for this disease. We began by qualitatively assessing the patterns of clustering of the disease incidence and risk factors, and the landscape level associations between ecosystems and the vectors. These analyses indicated that landscape alone does not explain the spatial distribution of ACL. Based on this information we proceeded with more quantitative analyses relating risk factors to the disease. Our more comprehensive analysis demonstrated that living close to the forest was negatively associated with infection incidence once social marginality was evaluated as a key variable in explaining disease pattern. The effects of these drivers are not monotonic, but rather display “breakpoints” or threshold values at which the shape and magnitude of the relationship change [15]–[17]. Forest cover certainly plays an important role in modulating the response of pathogen transmission to other environmental changes [18], specifically climate variability by the El Niño Southern Oscillation (ENSO). However, we have identified possible ecological mechanisms related to infection risk that may explain these macroscopic patterns, and suggest alternatives in planning development policies if the long term goals of biodiversity conservation, control of infectious diseases, and sustainable human well-being are to be pursued in concert.

## Materials and Methods

### Data

The monthly number of cases of American Cutaneous Leishmaniasis (ACL) from January 1996 through December 2000 was obtained from the epidemic surveillance service of Costa Rica “*Vigilancia de la Salud* ” for the 81 counties that comprise the country. The total number of cases for this period was 3379. County-level data on the percent of people living <5 km from the forest (%close) and percent forest cover, as of 2000, were obtained from [19]. Social marginality is in general referred as the lack or limited access to resources that ensure a satisfactory quality of life [20]. Social Marginalization index values (MI), based on the 2000 Costa Rican national census, were obtained from [20]. This marginalization index is a robust measure of social outcast status since it is constructed using several variables associated with social exclusion, including income, literacy, level of education, average distance to health centers, health insurance coverage, etc. Monthly rainfall data were obtained from 14 weather stations across the country available at the Earth Observing Laboratory, National Center for Atmospheric Research [http://data.eol.ucar.edu/], and the yearly average was calculated for each station. Ordinary kriging was employed to interpolate average rainfall values across the country using the Geostatistical Analyst extension in ArcGIS 9.1, and averages of mean, minimum (MinRflll) and maximum yearly rainfall for each county were calculated. An elevation data layer in raster format with 30 arc-second (∼1 km^2^) resolution was obtained from the United States Geologic Survey (USGS) [http://edc.usgs.gov/products/elevation/gtopo30/gtopo30.html], and minimum (ME), maximum, average and standard deviation of elevation for each county were calculated using Hawth's Analysis Tools for ArcGIS [21].

Data on species and locations of sand fly captures were obtained from systematic reviews on human biting species from Costa Rica [22],[23]. Coordinates of sand fly captures were compared against the Central American Ecosystems Map [http://mitchnts1.cr.usgs.gov/data/otheragency.html] created by Costa Rica's Centro Agronómico Tropical de Investigactión y Enseñanza (CATIE) and described by [24]. The ecosystem map, derived from Landsat satellite imagery, was created with ArcGIS at a resolution of 1 km^2^ grid cells and then used to define the ecological type in which each of the sand fly species was located.

## Statistical Methods

### Kuldorff's Scan Statistic

This method finds spatio-temporal clusters by detecting the excess of cases in a given region under the assumption that cases are generated by an inhomogeneous Poisson point process with an intensity, μ, proportional to the population at risk. The method is implemented by moving a circular window systematically through the study area, starting at the centroid of each location in the dataset [25]. The window expands to include the nearest region centroids, and its maximum size does not exceed 50% of the total population at risk size for the study period. The null hypothesis of a Poisson process is tested through a maximum likelihood ratio test that compares it to an alternative mod ,mel stating that this assumption is false, with the significance tested through multinomial Monte Carlo. The analysis was implemented with the Clusterseer software and significance of clusters was tested with 999 Monte Carlo randomizations. We assumed that the population at risk was that of the whole county, and used data from the 1983 and 2000 Costa Rican censuses [http://ccp.ucr.ac.cr/] with linear interpolation from January 1996 through December 2000.

### Local Indicators of Spatial Autocorrelation (LISA)

We used this technique to analyze the patterns of clustering in potential risk factors for the disease. LISA, a local adaptation of Moran's I, compares the value of the variable of interest in a given county with those in neighboring counties. The degree of similarity between neighboring counties was compared to that expected by chance to determine where clusters of high or low values occur [26]. To ensure the robustness of results, both queen contiguity and four-nearest neighbors were used as weights and the output compared for each variable using the GeoData Analysis software package.

### Negative Binomial Generalized Linear Models (NB-GLM) with breakpoints

We introduced breakpoints in predictors by transforming the predictor using a breakpoint basis function of the form:
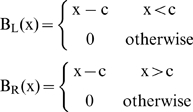
(1)where c is the breakpoint where the functions B_L_(x) and B_R_(x) join each other, and are used to separate the relationship between the response and the predictors to the left and the right of the break point respectively. This technique is known as hockey stick regression [27]. Four models where fitted using maximum likelihood for NB-GLM with logarithmic link and fixed over-dispersion parameter [28]. Nonlinear forms observed in the Generalized Additive Models (GAM) presented in Protocol S1 where approximated by using the following models:
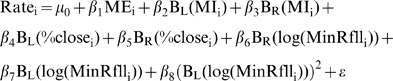
(2.1)

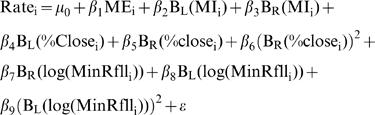
(2.2)

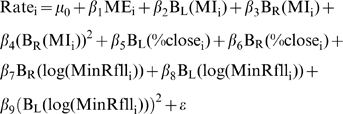
(2.3)

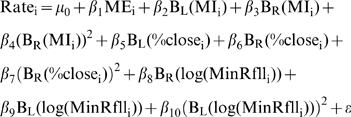
(2.4)


Models have the same predictors described for the GAM presented in Protocol S1. For the purpose of comparison a simpler null model without breakpoints was also fitted:
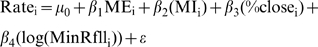
(3)as well as a model assuming smooth non-linear relationships with MI and %close:
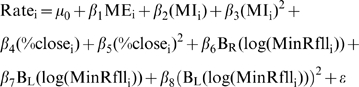
(4)


To make comparisons reliable, the variance over-dispersion parameter of the negative binomial response was fixed to 1, and not estimated independently for each model [26]. Models were fitted by off-setting the logarithm of population size on the right hand side of the equations as recommended for rate models [29]. Values for the break points “c” were estimated a priori by minimizing the value for Akaike Information Criterion (AIC) of a function fitting the model while considering breakpoint values for predictors: MI, %close and MinRfll using an algorithm based on the Newton method [30]. The model selected as “best” was further subjected to a process of model selection by backward elimination as described in [1],[27]. Goodness of fit for the final model was assessed using a Chi^2^ test with degrees of freedom (df) defined as n-p-1, where n is the number of observations, p the number of parameters estimated in the model, and the additional df accounts for the dispersion parameter of the negative binomial. Diagnostics for spatial autocorrelation were carried out by regressing residuals on the centroids of each county. The error (ε) was assumed to be identically and independently normally distributed for the linear predictor of the NB-GLM [28],[29].

### Linear Models and Analysis of Covariance (ANCOVA)

Parameters have a linear relationship with the response variable and were computed using ordinary least squares [27]. Models incorporated ENSO, county and their interaction as predictors. The definition for covariates and the response are similar to those used for the Linear Mixed Effects Models (LMEM), as are the assumptions about the error (ε, see Protocol S1). The linear model used for the ANCOVA is given by:
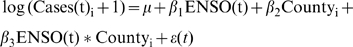
(5)


In the process of model building, autoregressive components were tested but they were not significant. However, for the sake of comparison, the fitting of model (5) only included the data from 1997 through 2000. Diagnostics for spatial autocorrelation were carried out by regressing residuals on the centroids of each county.

## Results

**Figure 1:**
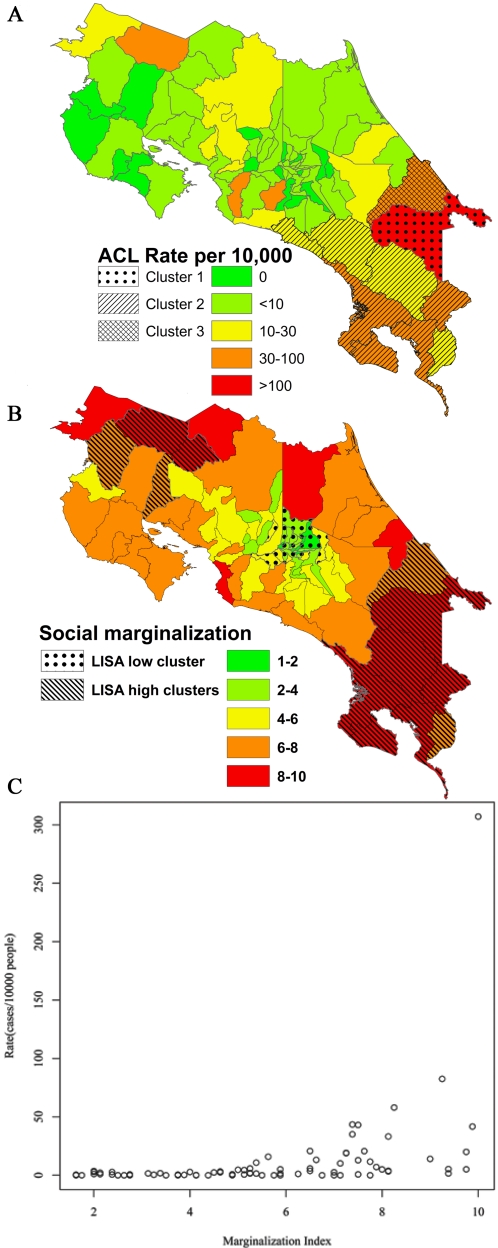
Patterns of clustering and Schmalhausen's law. (A) Quinquennial (1996–2000) cutaneous leishmaniasis case rates (cases/population) in Costa Rica at the county level. Colors indicate clustering in monthly rates per 10,000 inhabitants obtained using the Scan method: blue corresponds to the most likely cluster, comprised of the Talamanca county, with a monthly rate 308 per 10,000 from January 1999 to December 2000 (loglikelihood ratio = 3020.06, P<0.001); green depicts the second most likely cluster, comprised of the counties of Osa, Buenos Aires, Aguirre, Perez Zeledon, Golfito, Coto Brus, Aguirre y Corredores, with a rate of 7 per 10,000 from June 1996 to November 1999 (loglikelihood ratio = 515, P<0.001); and red corresponds to the third most likely cluster, comprised of the county of Limon with a rate of 12 per 10,000 from April 1997 to May 2000 (loglikelihood ratio = 265, P<0.001). (B) The county marginalization index (See Protocol S1 for details). Red and blue indicate clusters with high and low marginality, respectively, found using the LISA method with weights based on the 4 nearest neighbors (overall I = 0.7096, P<0.05). (C) County rate as a function of the marginalization index. Black dots represent counties with less than 2 cases in the five years. This pattern, which we call Schmalhausen's pattern, shows a significant positive correlation between marginality and the rate of the disease (r = 0.39, *t* = 3.8221, df = 79, P<0.0002), where a qualitative change in the relationship is apparent after and before a value of 4 in the marginalization index. Specifically, the variance increase for larger values of social marginalization, consistent with the prediction that new or anomalous conditions modify the system's sensitivity to other drivers.

We began by exploring whether the spatial distribution of disease incidence was heterogeneous across the country, a pattern that might be expected from the considerable heterogeneity of ecosystems in Costa Rica. Figure 1 shows that disease incidence and social marginalization (described in Materials and Methods) achieved their highest values in the same counties, a pattern not found for other ecological variables such as minimum rainfall, minimum elevation, landscape composition index, proportion of forest cover, or proportion of people living within 5 Km of the forest edge. This pattern was confirmed by spatial statistical analyses that detected overlapping geographical clusters for both disease and social marginalization, a pattern that was again absent for other variables and robust to the methodology applied to find clusters (Figure 1, Figure S1 and Figure S3). To further investigate counties where ACL was clustered, we analyzed the percentage of various landscape compositions using principal component analysis (PCA) for the most common landscape units known to harbor human biting sand fly species (Tables S1, S2, S3). No clear effect of landscape composition was found, as counties where the disease was clustered were within the ranges of variability of all counties in the country. We further tested the robustness of this result using multidimensional scaling, a method lacking the linearity constraints of PCA, with strikingly similar results (Tables S1, S2, S3 and Figure S2).

**Figure 2:**
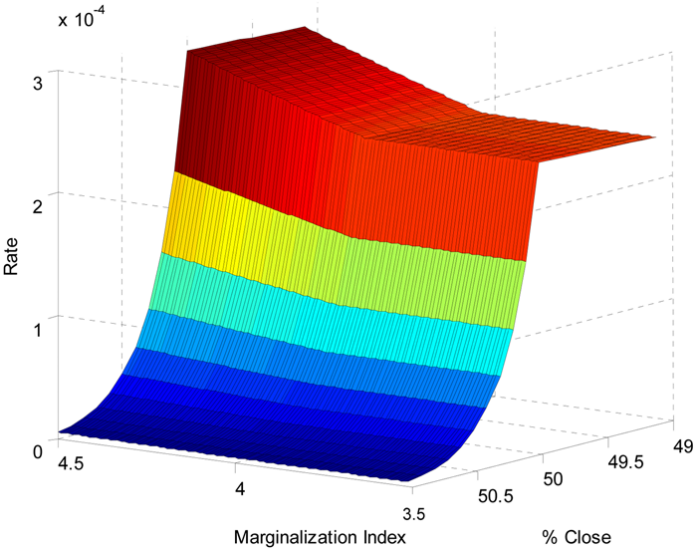
Breakpoints and discontinuous patterns of association. A schematic representation of the breakpoint in marginalization (MI) and people living close to the forest (%close), when minimum elevation (ME) is set to 500 m and rainfall (log(MinRfll)) is set at its breakpoint. The surface illustrates major qualitative differences in disease risk as a function of the covariates. Specifically, risk increases exponentially as the proportion of people living close to the forest decreases above the breakpoint. The change has the opposite sign and decreases in magnitude for smaller values below the breakpoint. Marginality exacerbates this difference above its own breakpoint. Parameters are those of the model selected as best. This model has 7 parameters (AIC = 5768.7) and fits the data satisfactorily (Residual deviance = 79.718, df = 72, P>0.24), explains 71.34% of the deviance (null deviance = 278.108) and is not different from the more complex models presented in Table 1, values for the coefficients are presented in Table S6.

To examine further and more quantitatively the factors determining observed spatial patterns of ACL, we fitted GAMs to the five-year ACL incidence rate (total cases during 1996 through 2000 divided by the 2000 population) as a function of several variables (see statistical methods in Protocol S1 for a detailed description and Table S4). A process of model selection by backward elimination (see Protocol S1) resulted in the following relevant variables: the marginalization index (MI), % of people living close to the forest (% close), log(minimum rainfall) and minimum elevation (ME). All variables except minimum elevation exhibited non-linear relationships with disease incidence, explaining 78% of the variance. Because GAMs are difficult to interpret and the fitted smoothed functions of GAMs showed clear qualitative changes (see Table S4 and Figure S3), we fitted somewhat simpler negative binomial generalized linear models (NB-GLM) that incorporated breakpoints (see Materials and Methods). The best model selected using this methodology accounted for 72% of variability (1- residual deviance /null deviance). Furthermore, major qualitative differences in the association of rates with some relevant variables were more easily visualized (Figure 2). Interestingly, a simpler model, not incorporating breakpoints, explained only 60 % of the variability (i.e. model deviance), and failed to capture the significance of the relationship between disease rates and proportion of people living <5 km from the forest border within each county. This breakpoint relationship with covariates was further supported by smaller Akaike Information Criterion (AIC) for breakpoint models, as compared to a model in which the relationships with covariates were described by smooth functions with the same number of parameters (second degree polynomial; Table 1).

**Table 1:** Breakpoint values for the natural logarithm of minimum rainfall, the marginalization index, and the percentage of people living close to the forest for the studied models.

Model	Log(Min Rainfall)	Margin Index	% Close	No. Parameters	AIC
I	7.78	4.13	49.40	8	567.7
II	7.78	4.13	49.98	9	569.5
III	7.77	4.13	49.99	9	569.1
IV	7.77	4.13	49.99	10	570.7
Smooth	7.79	Poly 2	Poly 2	8	574.2
Null	—	—	—	4	595.4

The number of parameters does not include the dispersion parameter for the negative binomial generalized linear models, which was set to 1 (see Protocol S1 for details).

**Figure 3:**
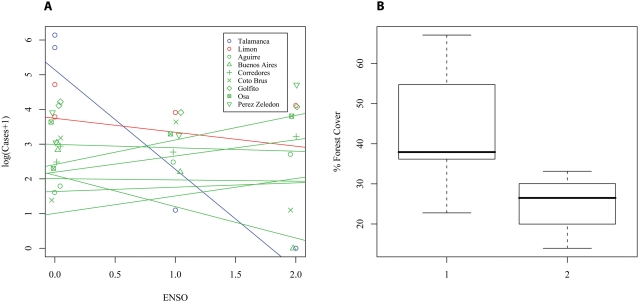
Cutaneous leishmaniasis in Costa Rica: Deforestation and El Niño Southern Oscillation (ENSO). (A) Local Effects of ENSO. Linear model results for a model testing for localized effects of ENSO in the counties where the disease was clustered. Color indicates clusters found with the spatio-temporal scan analysis of Figure 1, characters are used for the data in each individual county (For parameter values, see table in the appendix). For representation purposes, a small amount of noise was added in the x (ENSO) axis. The ANCOVA for this model showed the interaction of ENSO*County to be statistically significant (P<0.0113, for more details see Tables S7, S8). The model has a high goodness of fit (R^2^ = 0.85) that outperforms a similar model with the same number of parameters but that uses a first order autoregressive structure (R^2^ = 0.26) instead of ENSO. (B) Differences in forest cover for counties where the incidence diminishes or increases with ENSO. In the boxplot, 1 stands for the counties where the annual rate decreases with ENSO (Talamanca, Limón, Golfito, Buenos Aires & Coto Brus) and 2 for those where the incidence increases with ENSO (Aguirre, Corredores, Osa & Pérez-Zeledón). The difference is statistically significant as shown by a one tail Welch's t-test (a test robust to differences in variance) in which the alternative hypothesis is that the difference in forest cover between 1 and 2 is larger than 0 (*Welch's t* =  2.14, d.f. = 5.9, p<0.038).

To address effects of hierarchically nested geopolitical units (e.g., counties belonging to provinces) and of interannual climatic variability (El Niño Southern Oscillation (ENSO)), we fitted Linear Mixed Effects Models (LMEM). These models incorporated geopolitical subdivisions of the country as nested random factors, and ENSO as a continuous predictor (details in Protocol S1). Neither ENSO nor the geopolitical nesting of counties had significant effects based on bootstrap model comparisons, with the highest variability explained by unknown factors (Table S5). These results could indicate that the effects of ENSO were very local (county scale), and different across counties. To test the hypothesis of localized ENSO effects, we fitted an Analysis of Covariance (ANCOVA) to the counties where disease was clustered. The results showed a statistically significant interaction between ENSO and the considered counties (goodness of fit R^2^ = 85%). The effects of ENSO are variable, with some counties showing an increase and others a decrease in incidence during a cycle of the oscillation (Figure 3). The only variable that showed a significant difference between these two groups was the percentage of forest cover, with a significantly larger fraction (P<0.05) in counties where incidence decreased (Figure 3).

## Discussion

The finding that ACL tended to afflict socially marginal populations more heavily is common to other infectious diseases, and has been historically documented in public health studies particularly at small spatial scales [10],[31],[32]. We have shown here that social marginalization also can explain patterns of ACL at larger geographical scales. When this influence is taken into account, risk of infection is diminished among those living close to forests, an unexpected pattern in light of previous studies on the role of this habitat type. The pathway by which social marginalization promotes transmission of *Leishmania* in this context probably is linked to a major environmental problem affecting the tropics: destruction of forests and associated biodiversity. Forest clearing worldwide [33],[34], and especially in Costa Rica, is concurrent with development of large scale commercial agriculture [12],[13],[14], including monocultures of several commercial crops where ACL is clustered, and with accelerated human population growth [14]. This shift towards market-based agricultural production and rapidly expanding population is associated with new inequities in land tenure [35], increased numbers of landless peasants, and hence further pressure to cut down forests for local subsistence agriculture [13] and extraction of other natural resources [36],[37].

Risk of ACL infection in rural Costa Rica has been especially associated with the exposure to forests close to agricultural environments [38],[39]. The latter could imply that populations living inside or close to fragmented forests intermixed with crops where the overall biodiversity of the landscape is reduced could have a higher risk of infection when compared with those where the agricultural practices and crops allow the maintenance of biodiversity. Supporting this idea is the ecological knowledge about biodiversity in disturbed, fragmented, and isolated landscapes proceeds through a series of well-documented, ecological syndromes, starting with habitat destruction and associated biodiversity reduction [40], followed by loss of keystone species and resulting structural changes leading to reduced biodiversity [41]. Changes in biodiversity due to deforestation are probably of importance to ACL since the major reservoirs of *Leishmania* species are small mammals, including marsupials, rodents and sloths [5],[6]. Forest fragmentation has been shown to increase densities of these species, because in small and isolated habitat fragments, large predators are lost first, leading to major changes in inter-specific interactions that decrease mammal biodiversity and lead to the dominance of rodents [42],[43]. This scenario, extensively studied for Lyme disease which is another rodent-associated, vector-borne disease, involves increased diversity of hosts providing a “dilution” effect on transmission [43],[44]. Similar mechanisms may be at play for ACL as suggested by mathematical models of transmission dynamics and by field studies that show only a small number of mammal species are infected with *Leishmania* spp. among those that are frequently bitten by sand fly vectors [3],[45],[46].

Changes in landscape quality are also likely to affect composition of the arthropod vector community [47]. Interestingly, sand fly species richness is greater in traditional, shaded coffee agroecosystems than in those that are intensified and unshaded [48]. More generally, traditional coffee production supports similar biodiversity as undisturbed forests [49]. Reduced forest cover in our study modulated the effects of climate variability (ENSO), an interaction that may operate through multiple pathways. Increased temperatures in modified landscapes can directly affect transmission of vector-borne diseases [50]. The negative effects of climatic variability on crops, accompanied by associated increases in reliance on the exploitation of forest resources [1],[51], may have large impacts on the ACL transmission system. In addition, disruption of trophic structures known to increase densities of certain small mammals, including possible *Leishmania* spp. reservoirs [52]–[54], can be amplified by ENSO anomalies that alter resources [1],[52]. The influences of rainfall and elevation on the spatial distribution of ACL are probably mediated through the effects of humidity and temperature on the biology of both vectors and parasites [1].

Future work should examine the role of local climate variability encompassing multiple ENSO events over a longer time span, as was previously done at the coarse scale of the whole country [1]. A special emphasis should be put on elucidation of mechanisms acting at a local scale, since operational control strategies require further details about local characteristics increasing the risk of transmission, while always contextualizing these risk factors within the multidimensional nature of human disease. This can be achieved by considering aspects as diverse as the demographic structure of cases and the relationships between forest fragmentation and biodiversity on the endemic areas of the disease. Another effort could explore the relationship between ACL and different systems of agricultural production that might affect the ecology of transmission, as well as the perception and measures of protection that people take under different socio-economic conditions [10],[47],[48]. In a more theoretical realm, further attention should be given to a corollary of Schmalhausen's law of fundamental relevance to the resilience of ecosystems and their response to environmental change, namely the increase in the variance of systems under stress [e.g., 55]. Finally, our work underscores the need to place the control of ACL, and more generally of neglected tropical diseases and malaria, within a framework that encompasses ecologically sound development and viable solutions to the trade-offs between agriculture and conservation, such as shaded coffee production [48],[49],[51]. The quality of the landscape matrix is not only relevant to biodiversity conservation, as already recognized in studies of agroecosystems [37], but also to preventing the emergence and exacerbation of infectious diseases.

## Supporting Information

Table S1 **Ecosystems and number of locations where human biting sand fly species have been caught in Costa Rica (see references [22],[23] in the main article).**
(0.03 MB DOC)

Table S2 **Principal component analysis (PCA) for the landscape units where human biting sand flies have been caught in Costa Rica (see references [22],[23] in the main article).**
(0.03 MB DOC)

Table S3 **Factor loadings for ecosystems in components 1 and 2.**
(0.03 MB DOC)

Table S4 **Parameters, smooth function degrees of freedom, and significance for the GAM described in Equation 1.**
(0.03 MB DOC)

Table S5 **Comparison of Linear Mixed Effects models.**
(0.03 MB DOC)

Table S6 **Parameters for the model presented in Figure 2.**
(0.03 MB DOC)

Table S7 **Analysis of Covariance for the model in (5).**
(0.03 MB DOC)

Table S8 **Parameters for the linear model in (5).**
Intercept and ENSO are respectively the intercept and slope for Talamanca County, the reference county. For all other counties, intercept and slopes are found by adding the values in the table to the values for the reference county.
(0.04 MB DOC)

Protocol S1 **Additional statistical methods.**
(0.12 MB PDF)

Figure S1 (A) Weather stations and interpolated values. Clusters of deforestation: (B) Queen contiguity. (C) 4 nearest neighbors. (D) Ecosystems of Costa Rica and number of sand fly species for each locality (see references [22],[23] in the main article).
(0.83 MB TIF)

Figure S2 **Generalized Additive Model smooth functions.**
(A) Marginalization index. (B) % of People living within 5 km to the border of the forest. (C) Minimum elevation. (D) Log(minimum rainfall).
(0.71 MB TIF)

Figure S3 **Landscape dimension reduction.**
Top panels include the first three components from the PCA analysis presented in Tables S2 and S3. Bottom panels include three dimensions using a MDS analysis with 2.87 % for stress, a goodness of fit that is good.
(0.65 MB TIF)
