# Supplementary material for: Social Exclusion Modifies Climate and Deforestation Impacts on a Vector-Borne Disease
Source: PLoS Negl Trop Dis. 2008 Feb 6;2(2):e176. doi: 10.1371/journal.pntd.0000176 (PMC2238711; doi:10.1371/journal.pntd.0000176)
Supplement: Table S1 — Ecosystems and number of locations where human biting sand fly species have been caught in Costa Rica (see references [22],[23] in the main article). (0.03 MB DOC) [file pntd.0000176.s001.doc]

**Table S1** Ecosystems and number of locations where human biting sand fly species have been caught in Costa Rica (S6, S7)

| Locations | Ecosystem |
| --- | --- |
| 83 | Agriculture |
| 6 | Evergreen Tropical Forest |
| 1 | Deciduous Tropical Forest |
| 1 | Manglar Forest |
| 1 | Embalse |
