# Supplementary material for: Social Exclusion Modifies Climate and Deforestation Impacts on a Vector-Borne Disease
Source: PLoS Negl Trop Dis. 2008 Feb 6;2(2):e176. doi: 10.1371/journal.pntd.0000176 (PMC2238711; doi:10.1371/journal.pntd.0000176)
Supplement: Table S2 — Principal component analysis (PCA) for the landscape units where human biting sand flies have been caught in Costa Rica (see references [22],[23] in the main article). (0.03 MB DOC) [file pntd.0000176.s002.doc]

**Table S2** Principal Component Analysis (PCA) for the landscape units where human

biting sand flies have been caught in Costa Rica (S6, S7).

| Component | 1^st^ | 2^nd^ | 3^rd^ | 4^th^ |
| --- | --- | --- | --- | --- |
| Proportion of Variance | 0.64 | 0.26 | 0.09 | 0.01 |
| Cumulative Proportion | 0.64 | 0.90 | 0.99 | 1.00 |
