# Supplementary material for: Social Exclusion Modifies Climate and Deforestation Impacts on a Vector-Borne Disease
Source: PLoS Negl Trop Dis. 2008 Feb 6;2(2):e176. doi: 10.1371/journal.pntd.0000176 (PMC2238711; doi:10.1371/journal.pntd.0000176)
Supplement: Table S3 — Factor loadings for ecosystems in components 1 and 2. (0.03 MB DOC) [file pntd.0000176.s003.doc]

**Table S3** Factor Loadings for Ecosystems in components 1 & 2

| Component | 1^st^ | 2^nd^ |
| --- | --- | --- |
| Agriculture | 0.610 | --- |
| Evergreen Low-Lands | -0.247 | -0.880 |
| Evergreen Montane | -0.523 | 0.473 |
| Evergreen Sub-Montane | -0.541 | --- |
