# Supplementary material for: Social Exclusion Modifies Climate and Deforestation Impacts on a Vector-Borne Disease
Source: PLoS Negl Trop Dis. 2008 Feb 6;2(2):e176. doi: 10.1371/journal.pntd.0000176 (PMC2238711; doi:10.1371/journal.pntd.0000176)
Supplement: Table S4 — Parameters, smooth function degrees of freedom, and significance for the GAM described in Equation 1. (0.03 MB DOC) [file pntd.0000176.s004.doc]

**Table S4** Parameters, smooth function degrees of freedom and significance for the GAM described in equation 1.

| Parametric Coefficients | | | | |
| --- | --- | --- | --- | --- |
| Parameter | Estimate | S.E. | T | P |
| μ_0_ | -8.185 | 0.094 | -86.63 | <2e-16 |
| Smooth Terms | | | | |
| Variable | EDF | Rank | F | P |
| s(MI) | 3.504 | 8 | 3.345 | 0.03 |
| s(% Close) | 6.698 | 9 | 2.403 | 0.05 |
| s(ME) | 1.332 | 3 | 3.571 | 0.02 |
| s(log(MinRfll)) | 5.562 | 9 | 5.943 | 6E-05 |
