# Supplementary material for: Social Exclusion Modifies Climate and Deforestation Impacts on a Vector-Borne Disease
Source: PLoS Negl Trop Dis. 2008 Feb 6;2(2):e176. doi: 10.1371/journal.pntd.0000176 (PMC2238711; doi:10.1371/journal.pntd.0000176)
Supplement: Table S5 — Comparison of Linear Mixed Effects models. (0.03 MB DOC) [file pntd.0000176.s005.doc]

**Table S5** Comparison of Linear Mixed Effects models

| Models | Likelihood ratio test | Bootstrap P |
| --- | --- | --- |
| 6.1 and 6.2 | 0.3815 | 0.611 |
| 6.2 and 6.3 | 5.931e-08 | 0.437 |
| 6.3 and 6.4 | 9.920e-08 | 0.923 |
