# Supplementary material for: Social Exclusion Modifies Climate and Deforestation Impacts on a Vector-Borne Disease
Source: PLoS Negl Trop Dis. 2008 Feb 6;2(2):e176. doi: 10.1371/journal.pntd.0000176 (PMC2238711; doi:10.1371/journal.pntd.0000176)
Supplement: Table S6 — Parameters for the model presented in Figure 2. (0.03 MB DOC) [file pntd.0000176.s006.doc]

**Table S6**. Parameters for the model presented in Figure 2

| Parameter | Estimate | S. E. | *T* | P |
| --- | --- | --- | --- | --- |
| μ_0_ | -7.61828 | 0.471846 | -16.146 | < 2e-16* |
| β_1_ (ME) | -0.00157 | 0.000386 | -4.083 | 0.000112* |
| β_2_ (B_R_(MI)) | 0.375278 | 0.098044 | 3.828 | 0.000271* |
| β_3_ (B_R_(% Close)) | -3.93909 | 1.42758 | -2.759 | 0.007318* |
| β_4_ (B_L_(% Close)) | 0.019768 | 0.009035 | 2.188 | 0.031871* |
| β_5_ (B_L_(ln(MinRainfall))) | 2.434161 | 1.26397 | 1.926 | 0.058023 |
| β_6_ (B_L_(ln(MinRainfall))^2^) | -5.80016 | 1.202431 | -4.824 | 7.50E-06* |
| β_7_ (B_R_(ln(MinRainfall))) | 5.512966 | 1.726986 | 3.192 | 0.002083* |

*Statistically significant (P<0.05)
