# Supplementary material for: Social Exclusion Modifies Climate and Deforestation Impacts on a Vector-Borne Disease
Source: PLoS Negl Trop Dis. 2008 Feb 6;2(2):e176. doi: 10.1371/journal.pntd.0000176 (PMC2238711; doi:10.1371/journal.pntd.0000176)
Supplement: Table S7 — Analysis of Covariance for the model in (5). (0.03 MB DOC) [file pntd.0000176.s007.doc]

**Table S7** Analysis of Covariance for the model in (7)

| ANCOVA | | | |
| --- | --- | --- | --- |
| Factor | DF | F | P |
| Intercept | 1 | 311.541 | <0.0001 |
| County | 8 | 3.229 | 0.0891 |
| ENSO | 1 | 2.36 | 0.0621 |
| County*ENSO | 8 | 3.611 | 0.0113 |
| Error | 18 |  |  |
