# Supplementary material for: Social Exclusion Modifies Climate and Deforestation Impacts on a Vector-Borne Disease
Source: PLoS Negl Trop Dis. 2008 Feb 6;2(2):e176. doi: 10.1371/journal.pntd.0000176 (PMC2238711; doi:10.1371/journal.pntd.0000176)
Supplement: Table S8 — Parameters for the linear model in (5). Intercept and ENSO are respectively the intercept and slope for Talamanca County, the reference county. For all other counties, intercept and slopes are found by adding the values in the table to the values for the reference county. (0.04 MB DOC) [file pntd.0000176.s008.doc]

**Table S8** Parameters for the linear model in (7). Intercept and ENSO are respectively the

intercept and slope for Talamanca County, the reference county. For all other counties, intercept and slopes are found by adding the values in the table to the values for the reference county.

| Parameter | Estimate | Std. Error | *t* | P |
| --- | --- | --- | --- | --- |
| Intercept (Talamanca) | -4.5411 | 0.5007 | -9.07 | 3.92E-08 |
| ENSO (Talamanca) | -3.1518 | 0.4478 | -7.038 | 1.44E-06 |
| Aguirre | -3.6201 | 0.7081 | -5.113 | 7.28E-05 |
| Buenos Aires | -2.9878 | 0.7081 | -4.22 | 0.000515 |
| Corredores | -3.3055 | 0.7081 | -4.668 | 0.000191 |
| Coto Brus | -3.4213 | 0.7081 | -4.832 | 0.000134 |
| Golfito | -1.7606 | 0.7081 | -2.486 | 0.022943 |
| Limon | -2.6633 | 0.7081 | -3.761 | 0.00143 |
| Osa | -2.6661 | 0.7081 | -3.765 | 0.001417 |
| Perez Zeledón | -3.8459 | 0.7081 | -5.431 | 3.69E-05 |
| ENSO*Aguirre | 3.681 | 0.6333 | 5.812 | 1.66E-05 |
| ENSO*Buenos Aires | 1.7485 | 0.6333 | 2.761 | 0.01287 |
| ENSO*Corredores | 3.3862 | 0.6333 | 5.347 | 4.41E-05 |
| ENSO*Coto Brus | 2.737 | 0.6333 | 4.322 | 0.000411 |
| ENSO*Golfito | 3.0889 | 0.6333 | 4.877 | 0.000121 |
| ENSO*Limon | 3.0571 | 0.6333 | 4.827 | 0.000135 |
| ENSO*Osa | 3.5617 | 0.6333 | 5.624 | 2.46E-05 |
| ENSO*Perez Zeledón | 3.6914 | 0.6333 | 5.829 | 1.60E-05 |
