# Supplementary material for: Social Exclusion Modifies Climate and Deforestation Impacts on a Vector-Borne Disease
Source: PLoS Negl Trop Dis. 2008 Feb 6;2(2):e176. doi: 10.1371/journal.pntd.0000176 (PMC2238711; doi:10.1371/journal.pntd.0000176)
Supplement: Protocol S1 — Additional statistical methods. (0.12 MB PDF) [file pntd.0000176.s009.pdf]

Protocol S1: Additional Statistical Methods

Principal Component Analysis (PCA) and Multidimensional Scaling (MDS). These techniques were used to study the sand fly associated landscape composition of counties. Both PCA and MDS are dimension reduction techniques to identify main axes in the variability of multidimensional data sets. PCA is computed by finding the eigenvalues and respective eigenvectors of the variance-covariance matrix of a multidimensional dataset, producing scores (components) for each individual object in the dataset based on linear combinations of the variables [a1,a2]. In contrast, MDS is a different approach where distances among objects are computed using any of several different measures, returning coordinates for the points on the number of chosen dimensions for the analysis [a1]. For the MDS, Euclidean distances were used. Goodness of fit was measured using as a loss function the least squares on distances, or STRESS [a3]. For both techniques data on the proportion of county landscape cover associated to sand fly presence were normalized.

Generalized Additive Models (GAM). Additive models are a combination of parametric and non-parametric models [2,4]. Unlike linear models, where only linear parametric forms define the shape of the relationship between responses and predictors, GAM smooth functions, *s*(), that may be far from linear, can be used for such relationships. In this study, the smooth, non-parametric functions were computed using a penalized smoothing spline approach in which the parameters for smoothing were obtained by generalized cross-validation, using a function that weights the trade-off between the smoothing and the likelihood of the fitting [4]. An intensive process of model selection, based on the Akaike Information Criterion (AIC) and backward elimination [5], led to the selection of the following best model:

$$\mathrm{Rate_i} = \mu_0 + s(\mathrm{ME_i}) + s(\mathrm{MI_i}) + s(\mathrm{\%Close_i}) + s(\log(\mathrm{MinRfll_i})) + \varepsilon \qquad \text{(a.1)}$$

where $\mathrm{Rate_i}$ denotes the natural log of the five-year disease incidence rate (ln(cases/population size)), $\mathrm{ME_i}$, the minimum elevation, $\mathrm{MI_i}$, the marginalization index, $\mathrm{\%close_i}$, the percent of people living in a radius of 5 km to the forest edge, and $\mathrm{MinRfall_i}$, the natural log of average annual minimum rainfall. The index *i* indicates the county and ε is the

error which is assumed to be identical, independent and normal (i.e., $\varepsilon \sim N(0,\sigma^2)$). To handle the problem of logarithms for values of 0, we added 1 to all Rate values in (a.1). For this and subsequent models, unless otherwise indicated, all 81 counties were considered. Diagnostics for spatial autocorrelation were carried out by regressing residuals on the centroids of each county.

Linear Mixed Effects Models (LMEM). Mixed effects models can consider covariates as fixed effects or random effects. Fixed effects are unknown constants, while random effects are random variables [4]. As a result, a parameter for a fixed effect measures the mean effect in a response by unit or category change in a covariate, while for random effects they measure the variability due to a given covariate. We used these models to test for the spatial scale of highest variability in the political subdivisions of the country that included seven provinces subdivided in a total of 81 counties. For this analysis, we only considered the 59 counties where disease was present with >2 cases during the 5 year study period. Although this procedure may bias the results of the analysis, we considered it to be the most robust option to gain insights about the geopolitical scale of spatio-temporal variability in the data. This decision to only include a subset of the counties was taken because of the superior reliability of LMEM over their corresponding generalized versions, for which no maximum likelihood estimators have been derived [4], and the potential artifacts in the scales of variability due to the abundance of 0 values under the assumptions of linear models. The response in the model was the annual incidence (*Cases* in the models to follow), defined as the yearly total number of cases for a county, with the regression weighted by the total population in the county. These models were also used to test for an effect of the El Niño Southern Oscillation (ENSO) for the whole country by introducing a continuous predictor varying from 0 to 2, indicating the different phases of ENSO: 0 for years non-El Niño years (1997, 2000), 1 for the El Niño year (1998) and 2 for the year after this event (1999). Data from 1996 were excluded because they were lost as an autoregressive component in the response. This strategy was implemented to economize degrees of freedom (1 as opposed to the 3 needed by using a categorical predictor). Models were fitted by using restricted maximum likelihood estimators (REML) and compared through a parametric bootstrap [4]. Four models were considered:

$$\log(\text{Cases}(t)_{kj}+1)=\mu+\log(\text{population})+\phi\log(\text{Cases}(t-1)_{kj}+1)+\beta\text{ENSO}(t)+\sigma(t)_j+\delta(t)_{kj}+\varepsilon(t) \quad (a.2)$$

$$\log(\text{Cases}(t)_{kj}+1)=\mu+\log(\text{population})+\phi\log(\text{Cases}(t-1)_{kj}+1)+\sigma(t)_j+\delta(t)_{kj}+\varepsilon(t) \quad (a.3)$$

$$\log(\text{Cases}(t)_{k}+1)=\mu+\log(\text{population})+\phi\log(\text{Cases}(t-1)_{k}+1)+\delta(t)_{k}+\varepsilon(t) \quad (a.4)$$

$$\log(\text{Cases}(t)_{k}+1)=\mu+\log(\text{population})+\phi\log(\text{Cases}(t-1)_{k}+1)+\varepsilon(t) \quad (a.5)$$

The comparison between models a.2 and a.3 allows a test of the null hypothesis that ENSO has no effect at the country scale, and the comparison between models a.3 and a.4 for the null hypothesis that effects due to the geopolitical hierarchy (counties belonging to provinces) is irrelevant. The comparison between models a.4 and a.5 allows a test of the existence of a source of common variability across the counties or localized variability at the county level. Models are autoregressive (ϕ), with σ and δ representing province and county level variability, *t* the time, *k* the county, and *kj* the county *k* belonging to province *j*. σ, δ, ε are assumed to be i.i.d. normal. Diagnostics for spatial autocorrelation were carried out by regressing residuals on the centroids of each county.

References

1. Borg I, Groenen P (1997) Modern multidimensional scaling: theory and applications. New York: Springer-Verlag.
2. Venables WN, Ripley BR (2002) Modern Applied statistics with S. New York: Springer-Verlag.
3. Kruskal JB (1964) Multidimensional scaling by optimizing goodness of fit to a nonmetric hypothesis. Psychometrika. 29: 1-27.
4. Faraway JJ (2005) Extending the linear model with R: generalized linear, mixed effects and nonparametric regression models. Boca Raton: Chapman Hall/ CRC.
5. Faraway JJ (2005) Linear Models with R. Boca Raton: Chapman Hall/ CRC.
