# Supplementary figures and images for: Social Exclusion Modifies Climate and Deforestation Impacts on a Vector-Borne Disease
Source: PLoS Negl Trop Dis. 2008 Feb 6;2(2):e176. doi: 10.1371/journal.pntd.0000176 (PMC2238711; doi:10.1371/journal.pntd.0000176)

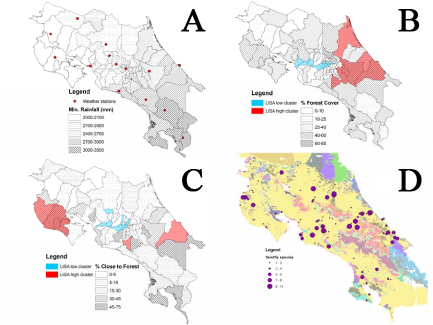

Supplement: Figure S1 — (A) Weather stations and interpolated values. Clusters of deforestation: (B) Queen contiguity. (C) 4 nearest neighbors. (D) Ecosystems of Costa Rica and number of sand fly species for each locality (see references [22],[23] in the main article). (0.83 MB TIF) [file pntd.0000176.s010.tif]

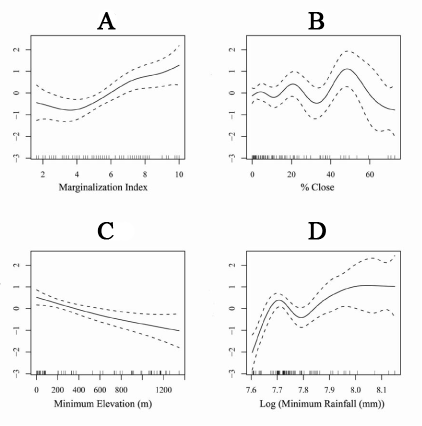

Supplement: Figure S2 — Generalized Additive Model smooth functions. (A) Marginalization index. (B) % of People living within 5 km to the border of the forest. (C) Minimum elevation. (D) Log(minimum rainfall). (0.71 MB TIF) [file pntd.0000176.s011.tif]

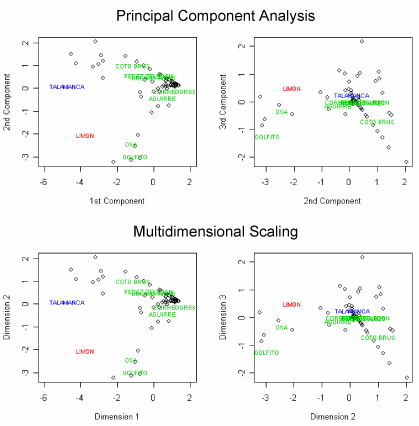

Supplement: Figure S3 — Landscape dimension reduction. Top panels include the first three components from the PCA analysis presented in Tables S2 and S3. Bottom panels include three dimensions using a MDS analysis with 2.87 % for stress, a goodness of fit that is good. (0.65 MB TIF) [file pntd.0000176.s012.tif]
